# Facial Expressions and Emotion Labels Are Separate Initiators of Trait Inferences From the Face

**DOI:** 10.3389/fpsyg.2021.749933

**Published:** 2021-12-08

**Authors:** Anthony Stahelski, Amber Anderson, Nicholas Browitt, Mary Radeke

**Affiliations:** ^1^Department of Psychology, Central Washington University, Ellensburg, WA, United States; ^2^Department of Psychology, Universitetet i Oslo, Oslo, Norway

**Keywords:** facial expressions, emotion labels, facial inferencing, valence, personality

## Abstract

Facial inferencing research began with an inadvertent confound. The initial work by Paul Ekman and Wallace Friesen identified the six now-classic facial expressions by the emotion labels chosen by most participants: anger, disgust, fear, happiness, sadness, and surprise. These labels have been used by most of the published facial inference research studies over the last 50 years. However, not all participants in these studies labeled the expressions with the same emotions. For example, that some participants labeled scowling faces as disgusted rather than angry was seen in very early research by Silvan Tomkins and Robert McCarty. Given that the same facial expressions can be paired with different emotions, our research focused on the following questions: Do participants make different personality, temperament, and social trait inferences when assigning different emotion labels to the same facial expression? And what is the stronger cause of trait inferences, the facial expressions themselves, or the emotion labels given to the expressions? Using an online survey format participants were presented with older and younger female and male smiling or scowling faces selected from a validated facial database. Participants responded to questions regarding the social traits of attractiveness, facial maturity, honesty, and threat potential, the temperament traits of positiveness, dominance, excitability, and the Saucier Mini-marker Big Five personality trait adjective scale, while viewing each face. Participants made positive inferences to smiling faces and negative inferences to scowling faces on all dependent variables. Data from participants labeling the scowling faces as angry were compared to those who labeled the faces as disgusted. Results indicate that those labeling the scowling faces as angry perceived the faces significantly more negatively on 11 of the 12 dependent variables than those who labeled the same faces as disgusted. The inferences made by the “disgust” labelers were not positive; just less negative. The results indicate that different emotion labels made to scowling faces can either intensify or reduce negativity in inferences, but the facial expressions themselves determine negativity or positivity.

## Introduction

Facial expressions and the emotional perceptions made from those expressions have been confounded from the earliest facial inferencing studies. The facial expression stimuli perceived as happy, angry, fearful, disgusted, sad, or surprised have been consistently identified by these emotion labels (Ekman and Friesen, 1971). However, facial expressions and emotional interpretations (labels) are not the same thing. From the perceiver standpoint, facial expressions are physical stimuli on the faces of perceived persons. The emotional interpretations of the expressions are inferential labels made by perceiving persons about internal states of the perceived.

If participants in facial inferencing studies label these facial expressions with the expected emotion label (for example, a smiling face labeled as happy), the labeling was listed as “accurate” or “correct,” because the labels coincided with the original Ekman and Friesen pairings of facial expressions and emotion labels. However, even in the earliest studies, some participants made unexpected emotional inferences of facial expressions, such as labeling a scowling face as disgusted (Tompkins and McCarter, 1964). The emotional mislabeling of faces (according to the original Ekman and Friesen facial expression-emotion label pairings) continues to occur in facial inferencing research (Widen and Russell, 2008). The mislabeling almost always occurs between faces showing the negative emotions: anger, disgust, fear, and sadness, with the highest frequency of mislabeling occurring between anger and disgust (Egger et al., 2011).

Separating facial expressions from perceivers’ emotion labels lead to a question: Does the facial expression stimulus or the emotion label perception have more effect on trait inferences made from faces? Previous research has supported each factor. Sauter et al. (2011) demonstrated that facial inferences can be made from facial expressions alone, regardless of emotion labels or lexical context. In contrast studies by Tiedens (2001), Gendron et al. (2012) and Fugate et al. (2018) demonstrate that varying emotion labels to the same expressions can shift facial inferences. Clearly both facial expressions themselves and the attached emotion labels are determinants of facial inferencing. Further research is needed to determine when and how each factor influences the inference process.

The concepts of emotional valence and arousal may help to disentangle the inference influence of facial expressions versus emotion labels. Valence and arousal are two of the core dimensions of an emotional experience (Barrett, 1998), and Mehu and Scherer (2015) demonstrated that both valence and arousal influence the classification of emotional facial expressions into discrete categories.

Emotional valence was initially defined as either a positive (pleasant) or negative (unpleasant) affective response to an external or internal stimulus (Lewin, 1951). Since then the concept of emotional valence has become essential to defining emotional experience (Solomon and Stone, 2002; Colombetti, 2005; Fridja and Scherer, 2009). Experiencing emotional valence does not require the recognition of a specific emotion, since perceiving a stimulus as either positive or negative can initiate a motivational and behavioral sequence (Barrett, 2006). Support for the idea that valence can be experienced without specific emotion labeling comes from two studies that have examined emotional valence and physiologic and brain activity. Anders et al. (2004) assessed participant verbal and physiologic reactions and brain activity, while they viewed pictures of pleasant and unpleasant events. Their results indicated that different brain mechanisms and pathways (specifically relating to the amygdala, anterior parietal cortex, left supramarginal gyrus, and insular cortex) underlie the physiologic and verbal responses participants made to either pleasant or unpleasant stimuli. Viinikainen et al. (2010) also exposed participants to either pleasant or unpleasant pictures and discovered that different brain mechanisms and pathways were specifically associated with either pleasant or unpleasant pictures.

Shuman et al. (2013) presented a multi-faceted approach to valence, in which they distinguished between macro and micro-valences. Macro-valence derives from the basic pleasant/positive and unpleasant/negative distinction discussed above, and micro-valences, which are evaluations of various internal characteristics of the perceived individual and various environmental circumstances. Shuman et al. specify four micro-valences, one of which, goal conduciveness, is relevant to the emotion labeling and trait inferencing process. Goal conduciveness is defined as a perceiver appraisal of a situation in terms of the perceiver’s need satisfaction or goal achievement. According to the theory, faces would be evaluated as either pleasant or unpleasant, and then more specifically in terms of the perceiver’s needs and goals. It is possible that facial expressions themselves initiate macro-valence and that specific emotion labels result from consideration of various micro-valences.

Presumably arousal as well as valence plays a role in the facial inference process. Emotional arousal is defined as the level of activation or excitement that an individual experiences in an emotional episode (Rule and Nesdale, 1976). Arousal can be measured physiologically *via* heart rate and numerous other measures, and it can be assessed by self-report (Barrett et al., 2004). Although valence and arousal are considered to be separate variables influencing emotional experience, researchers have examined their interaction. Kuppens et al. (2013) reviewed this research across a variety of emotional experiences, assessing whether there was any discernable pattern between valence and arousal. The results of some reviewed studies revealed a V-shaped pattern shown on a two-dimensional graph, with arousal on the Y axis and valence on the X axis. The valence scaling went from intensely negative through neutral to intensely positive. Arousal went up as reported valence approached either valence extreme. Support for the V-shaped valence-arousal model comes from studies by Jallais and Gilet (2010) and Kuhbandner and Zehetleitner (2011) where arousal increased with the presentation of more extremely valent stimuli, such as an extremely angry face versus a mildly angry face.

The purpose of this study is an attempt to further understand the inferential impact (personality, temperament, and social traits) of facial expressions versus the inferential impact of the emotion labels attached to the facial expressions, in the context of valence and arousal. As stated above, perhaps, facial expressions themselves initiate the macro-valence of pleasantness or unpleasantness, and the emotion labels attached to the expressions reflect different micro-valent evaluations and differential arousal which then initiate specific emotion-related trait inferences. In this study, if the participants perceive the same scowling expressions as disgusted rather than angry, does this further change the personality and social inferences beyond those that are made to the facial expression itself? Two experiments address this question. In Experiment 1, we compared participant inferences to scowling and smiling faces, and in Experiment 2, we compared participants who labeled the scowling faces as angry versus those who labeled the same scowling faces as disgusted. We hypothesize that scowling faces will initiate negative macro-valence and overall negative inferences compared to the positive macro-valence initiated by smiling faces, regardless of the emotion label assigned to the faces (Experiment 1, Hypothesis 1). We further hypothesize that the anger emotion label made to the scowling faces as opposed to the disgust label will result in more negative inferences (Experiment 2, Hypothesis 2). We also hypothesize that participant reports of the pictured models’ arousal will be greater for those participants who labeled the smiling faces as happy, and the scowling faces as angry rather than disgusted (Experiment 1; Experiment 2, Hypothesis 3).

## Experiment 1 – Materials and Methods

### Participants

The data for this experiment were a compilation of eight different data sets, collected over a two-week period in 2017. Participants from the US were recruited using the MTurk crowdsourcing research service. The total number of participants was recruited for the study was 2,881. Due to incomplete responses, not all participant data are included in this study and we have indicated the loss of participant data in each section. Racial demographics indicated that participants were predominantly white (73%), followed by Asian American (10%), African American (9%), Hispanic or Latino (6%), Native Hawaiian or Pacific Islander (0.1%), and Multi- and Bi-racial (1%). Two percent of participants did not indicate race. Age demographics ranged from 21 to 51 with most participants being between 26 and 30 years of age. Fifty-one percent of participants identified as female and 48% identified as male, the remainder identifying as neither male nor female.

### Photographic Stimuli and Measures

#### Facial Photographs

**Figure 1:**
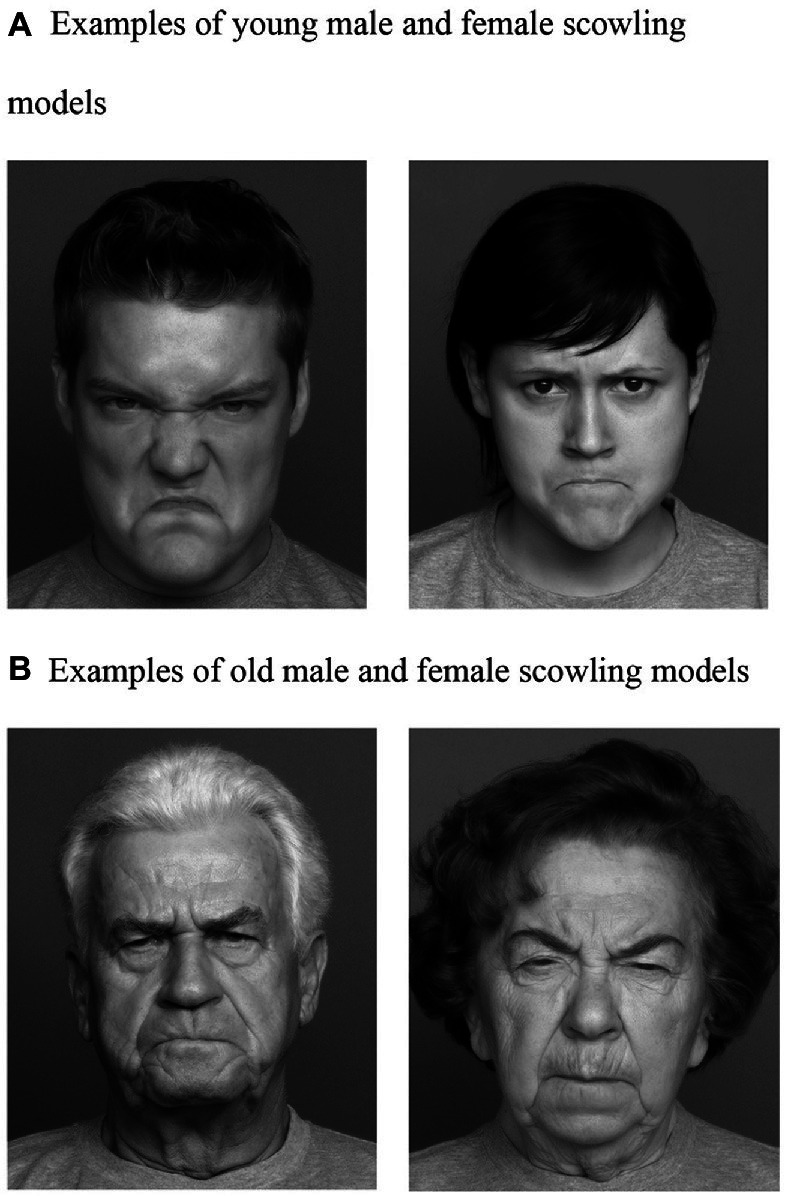
Facial photographs (Ebner et al., 2010) the Center for Lifespan Psychology, Max Planck Institute for Human Development, Berlin, Germany. Used with permission from the Max Planck Institute. These models are examples of images used in this study and not the actual images used. **(A)** Examples of young male and female scowling models. **(B)** Examples of old male and female scowling models.

The eight pairs of photographs (two older and younger scowling male and female, and two older and younger smiling male and female models) selected for use in this study were from the FACES database created by the Max Plank Institute and used with permission from the Center for Lifespan Psychology, Max Planck Institute for Human Development, Berlin, Germany (see Figure 1 for examples of the scowling faces). Criteria for selecting these eight pairs of photographs were based on the frequency of facial expression identification (Ebner et al., 2010). The scowling and smiling faces were identified by Ebner et al. (2010) as displaying the emotions of anger or happiness, respectively. Overall, the frequency of emotion labels for the facial expression photographs ranged from 96 to 68% (see Ebner et al., 2010 for a full explanation of the validation procedure and results).

The data for this study were originally intended for studies that included comparisons of the age and gender of the facial models; however, this study does not include such comparisons. Additionally, due to the unequal sample sizes, an analysis of model gender and age would have resulted in even greater disparity between the sample sizes.

#### The Big Five Personality Traits: Mini-Markers

To assess personality trait inferences, the 40 adjective Mini-marker Scale (Saucier, 1994, 2002), derived from an original set of 100 adjective markers developed by Goldberg (1992), was used to provide a measure for the Big Five personality traits (*Agreeableness, Conscientiousness, Emotional Stability, Extraversion, and Openness*). Forty different adjectives were presented to participants who were asked how much or how little each of those adjectives applies to the photographed person on an eight-point Likert-type scale, from 1(extremely inaccurate) to 8 (extremely accurate). Each of the Big Five traits is represented by eight adjectives, according to the validated mini-marker subset created by Saucier (1994, 2002). This subset was created because the original 100 adjective set was often not practical when used with other assessment tools. For comparison, Cronbach’s alpha coefficients for the Goldberg scale and the Mini-marker scale can be found in Radeke and Stahelski (2020). Following data collection, the forty adjectives were collapsed into the five corresponding factors for analysis. Negative adjectives, such as “disorganized,” which indicates a lack of conscientiousness, were reverse scored (Saucier, 1994). Participants who failed to complete at least five consecutive Mini-marker adjectives were excluded from the analysis, resulting in the exclusion of 439 participants from the total sample.

#### Self-Assessment Manikin

Using a Likert-type scale, the Self-assessment Manikin (SAM) is a semantic differential temperament pictorial scale measuring the extent to which participants found images to be positive, dominant, and arousing (Bradley and Lang, 1994). This scale required participants to rate each photograph for *Positivity/Negativity* (1 = extremely positive to 9 = extremely negative), *Subordinate/Dominant* (1 = extremely subordinate to 9 = extremely dominant), and *Calm/Excited* (1 = extremely calm to 9 = extremely excited). All but two participants completed the SAM question set.

#### Social Perceptions

Using a Likert-type scale, participants were asked to answer four questions addressing the following social perceptions: *attractiveness* (1 = extremely unattractive to 7 = extremely attractive); *facial maturity* (1 = extremely baby-faced to 5 = extremely mature-faced); *honesty* (1 = extremely dishonest to 7 = extremely honest); and *threatening* (1 = extremely non-threatening to 7 = extremely threatening). These questions were presented for each photograph. All participants completed the Social Perception question set.

### Procedure

Participants were recruited through Amazon’s MTurk service, and then directed to an online Qualtrics survey, once they agreed to participate in the study. Participants were shown two randomly presented facial expressions (one smiling and one scowling) of one of the eight photographed models. After participants viewed the facial photograph, they were asked to identify the emotion displayed by the model in the photograph (forced choice; angry, disgusted, fearful, happy, sad, and surprised) and were asked to answer questions about the characteristics of the person in the photograph, while the photograph was still on the screen. The Mini-marker 40 adjectives question set, SAM, and the Social Perception question set were presented in a random order for each participant. Average completion time of the assessment was 25 min. Prior to participating in the online survey, informed consent was obtained from all participants. This study was approved by the authors’ Institutional Review Board, Human Subjects Review Council and was conducted according to the principles expressed in the Declaration of Helsinki.

### Statistical Analyses

Manova and univariate analyses were conducted using the open access statistical package, Jamovi 2.0.1. Additionally, tests for homogeneity (*Box’s M* and *Levene’s test*) were conducted. In cases where violations of homogeneity were observed, a correction using a robust independent *t*-test (*Yuen’s t*-test with bootstrapping; Luh and Guo, 2007; Erceg-Hurn and Mirosevich, 2008) was employed. A more conservative alpha level of 0.01 was also used for Manova and Anova and *t*-test, regardless of the violations of homogeneity.

## Experiment 1 – Results

### Combination of Data Sets

In order to combine the data for analysis, we needed to insure the homogeneity of the data sets. Using, an 8 (Data Set) × 2 (Reported Emotion; Angry and Disgusted) Manova and univariate analyses were conducted for each of the three dependent variable groups. Manova analyses revealed non-significant interactions of Data Set × Reported Emotion for the Mini-markers [*Pillai’s Trace* = 0.03, *F*(35, 5,655) = 1.08, *p* = 0.35], SAM, [*Pillai’s Trace* = 0.06, *F*(21, 4,248) = 1.38, *p* = 0.114], and Social Perception variables [*Pillai’s Trace* = 0.02, *F*(28, 5,664) = 0.98, *p* = 0.49]. For the dependent variable groups of Mini-markers and SAM, the assumption of homogeneity was maintained (Box’s M test *p* > 0.05). Based on these non-significant findings, we felt confident that the data sets were similar enough to warrant combing for the overall analysis.

### Overall Comparison of Inferences Made to Smiling and Scowling Faces

A comparison was conducted of the participants’ inferences made to the scowling and smiling faces to test Hypothesis 1 that scowling faces will initiate negative macro-valence and overall negative inferences compared to smiling faces, regardless of the emotion label assigned. Differences between inferences made to the scowling and smiling faces were significant for the five Mini-markers traits [scowling *N* = 1,208, smiling *N* = 1,234; *Pillai’s Trace* = 0.65, *F*(5, 2,436) = 885, *p* < 0.001, *Box’s M homogeneity test* = 645(15), *p* < 0.001], the Social Perception traits [scowling *N* = 1,497, smiling *N* = 1,384; *Pillai’s Trace* = 0.60, *F*(4, 2,876) = 1,076, *p* < 0.001, *Box’s M homogeneity test* = 201(10), *p* < 0.001], and the SAM [scowling *N* = 1,495, smiling *N* = 1,384; *Pillai’s Trace* = 0.63, *F*(3, 2,875) = 1,605, *p* < 0.001, *Box’s M homogeneity test* = 212(6), *p* < 0.001]. As can be seen in Tables 1–3, inferences made to the scowling faces were less *Attractive*, *Honest*, more *Facially Mature* and more *Threatening*, more *Negative*, less *Calm*, and more *Dominant* than smiling faces (*p* < 0.001), regardless of the perceived emotion label. Additionally, the scowling face was perceived as less *Agreeable*, *Conscientious*, *Emotionally Stable*, *Extraverted*, and *Open* than the smiling face (*p* < 0.001), regardless of the perceived emotion label. *Levene’s test for homogeneity* revealed *p* > 0.05 for each of the Mini-markers, Social Perception, and SAM variables. A robust *independent t*-test was performed (*Yuen’s* bootstrapped 1,000 samples, trim portion set to 0.2). See Table 4 for a summary of the robust *independent t*-tests.

**Table 1:** Overall comparison of mini-marker inferences made to smiling and scowling faces, *M* (*SD*).

	Scowl	Smile
	*M(SD)*	*M(SD)*
Agreeableness	3.11(1.36)	6.35(1.10)
Conscientiousness	4.23(1.06)	5.64(1.02)
Emotional Stability	3.28(1.12)	5.80(1.14)
Extraversion	5.08(0.94)	5.86(0.98)
Openness	3.86(111)	5.24(1.05)

*Agreeableness (1 = disagreeable; 8 = agreeable); Conscientiousness (1 = unconscientious; 7 = conscientious); Emotional stability (1 = high emotional stability; 8 = low emotional stability); Extraversion (1 = introversion; 8 = extraversion); and Openness (1 = closed; 8 = open)*.

**Table 2:** Overall comparison of sam inferences made to smiling and scowling faces, *M* (*SD*).

	Scowl	Smile
	*M (SD)*	*M (SD)*
Positive/Negative^a^	7.44(1.96)	2.44(2.08)
Subordinate/Dominant^b^	6.62(1.99)	5.1(1.70)
Calm/Excited^c^	6.81(2.06)	4.97(2.23)

a *Positive/Negative (1 = positive; 9 = negative)*.

b *Subordinate/Dominant (1 = subordinate; 9 = dominant)*.

c *Calm/Excited (1 = calm; 9 = excited)*.

**Table 3:** Overall comparison of social perception inferences made to smiling and scowling faces, *M* (*SD*).

	Scowl	Smile
	*M (SD)*	*M (SD)*
Attractive	3.16(1.51)	4.87(1.27)
Facial Maturity	3.77(1.24)	3.18(1.31)
Honest	4.78(1.22)	6.3(1.0)
Threat	5.02(1.46)	1.94(1.13)

*All variables scored as 1 (lowest) and 7 (highest)*.

**Table 4:** Yuen’s robust independent samples *t*-test, scowl and smile.

	*t*	Bootstrapped *t*	*df*	*p*	*Mean diff Scowl - Smile*
Agreeable	58.8	−58.7	1,398	< 0.001	−3.51
Conscientiousness	30.0	−29.9	1,412	< 0.001	−1.39
Emotional stability	50.1	−50.0	1,464	< 0.001	−2.65
Extraversion	19.9	−19.9	1,423	< 0.001	−0.87
Openness	30.7	−30.6	1,424	< 0.001	−1.40
Attractiveness	31.8	−31.7	1,594	< 0.001	−1.98
Facial Maturity	12.4	12.4	1,712	< 0.001	0.93
Honesty	34.7	−34.6	1,693	< 0.001	−1.66
Threat	90.2	90.2	1,525	< 0.001	3.63
Positive/Negative	99.6	99.6	1,642	< 0.001	6.31
Subordinate/ Dominant	24.6	24.6	1725	< 0.001	1.78
Calm/Excited	22.5	22.5	1,684	< 0.001	2.18

## Experiment 2 – Materials and Methods

### Participants

The data for Experiment 2 were a subset of the data set used in Experiment 1. Only the scowling face was used for Experiment 2 and only instances when the face was identified with the emotion label of anger or disgust. Refer to the Results section for total number of participants for each analysis.

### Photographic Stimuli and Measures

#### Facial Photographs

The facial photographs used in Experiment 2 included only the scowling faces (anger and disgust) from the FACES database, see Figure 1.

#### The Big Five Personality Traits: Mini-Markers

See “Experiment 1 The Big Five Personality Traits: Mini-Markers” for a description of this measure.

#### Self-Assessment Manikin

See “Experiment 1 Self-Assessment Manikin” for a description of this measure.

#### Social Perceptions

See “Experiment 1 Social Perceptions” for a description of this measure.

### Procedure

The procedure for Experiment 2 was the same as Experiment 1.

## Experiment 2 – Results

### Emotion Label Analysis

While the accurate emotion label of the scowling face was anger and the smiling face was happy (based on the FACES database identification, Ebner et al., 2010), participants were not always accurate when labeling the faces. Participants identified the scowling faces as fearful (2%), sad (2%), disgusted (11%), and angry (85%). The smiling faces were always identified as happy (100%). The following sections present all analyses using Manova and univariate analyses to assess the Mini-marker, SAM, and Social Perception dependent variables for those participants who labeled the scowling faces as either angry or disgusted.

### Mini-Marker Traits; Scowling Faces Labeled as Anger and Disgust Only

A comparison of the scowling faces was done to assess the hypothesis that the different emotion labels (micro-valences) made to the scowling faces will result in differences in the negativity of the inferences (Hypothesis 2). The analyses in the following sections focus only on the scowling faces labeled as angry or disgusted.

The overall Manova for the Mini-marker question set was significant [*N* = 1,147; *Pillai’s Trace* = 0.07, *F*(5, 1,141) = 16.5, *p* < 0.001, *Box’s M test for homogeneity* = 38.9(15), *p* < 0.001]. Anovas revealed the emotion labels of anger and disgust altered the perceptions of *Agreeableness* [*F*(1, 1,145) = 73.53, *p* < 0.001, *n^2^ = 0*.06), *Conscientiousness* [*F*(1, 1,145) = 12.60, *p* < 0.001, *n^2^ = 0*.01], *Emotional Stability* [*F*(1, 1,145) = 32.20, *p* < 0.001, *n^2^ = 0*.03], *Extraversion* [*F*(1, 1,145) = 9.24, *p* = 0.002, *n^2^ = 0*.008], and *Openness* [*F*(2, 1,145) = 19.7, *p* < 0.001, *n^2^ = 0*.02]. *Levene’s test for homogeneity* revealed *p* > 0.05 for each of the Mini-markers; therefore, a robust *independent t*-test was performed (*Yuen’s* bootstrapped 1,000 samples, trim portion set to 0.2). Participants who labeled the scowling faces as angry, perceived the faces as less *Agreeable* [*Yuen’s bootstrapped t*(94.9) = −7.09, *p* < 0.001, *MD* = −1.03], less C*onscientious* [*Yuen’s bootstrapped t*(96.2) = −2.57, *p* = 0.012, *MD* = −0.28], less *Emotionally Stable* [*Yuen’s bootstrapped t*(106.2) = −5.59, *p* < 0.001, *MD* = −0.61], more *Extraverted* [*Yuen’s bootstrapped t*(111.3) = 2.54, *p* = 0.013, *MD* = 0.20], and less *Open* [*Yuen’s bootstrapped t*(101.2) = −3.57, *p* < 0.001, *MD* = −0.42] than those who labeled the faces as disgusted.

### Self-Assessment Manikin; Scowling Faces Labeled as Anger and Disgust Only

The overall Manova for the SAM question set was significant [*N* = 1,432; *Pillai’s Trace* = 0.05, *F*(3, 1,428) = 26.1, *p* < 0.001, *Box’s M test for homogeneity* = 11.6(6), *p* = 0.07]. Levene’s test for homogeneity revealed *p* > 0.05 for each of the SAM variables; therefore, no adjustments were made with the exception of the use of a more conservative alpha (*p* < 0.01). Anovas revealed that the emotion labels of anger and disgust altered the perceptions of *Positivity* [*F*(1, 1,430) = 22.7, *p* < 0.001, *n^2^ = 0*.02], *Dominance* [*F*(1, 1,430) = 33.5, *p* < 0.001, *n^2^ = 0*.02], and *Arousal or Excited* [*F*(1, 1,430) = 55.5, *p* < 0.001, *n^2^ = 0*.04]. Participants who labeled the scowling faces as angry perceived the faces to be more *Negative* [*MD* = 0.75, *t*(1430) = 4.76, *p* < 0.001], more *Dominant* [*MD* = 0.92, *t*(1430) = 5.79, *p* < 0.001], and more *Aroused* or *Excited* [*MD* = 1.21, *t*(1430) = 7.45, *p* < 0.001] than those who labeled the faces as disgusted.

### Social Perceptions; Scowling Faces Labeled as Anger and Disgust Only

The overall Manova for the Social Perception question set was significant [*N* = 1,432; *Pillai’s Trace* = 0.05, *F*(4, 1,427) = 18.70, *p* < 0.001, *Box’s M test for homogeneity* = 8.54(10), *p* = 0.58]. Anovas revealed that the emotion labels of anger and disgust altered the social perceptions of *Honesty* [*F*(1, 1,430 = 13.38, *p* < 0.001, *n^2^* = 0.009] and *Threat* [*F*(1, 1,430) = 52.11, *p* < 0.001, *n^2^* = 0.03]. The emotion labels did not alter the social perceptions of *Attractiveness* and *Facial Maturity* (*p* > 0.05). *Levene’s test for homogeneity* revealed *p* < 0.05 for the variables *Honesty* and *Threat*; therefore, a robust *independent t*-test was performed (*Yuen’s bootstrapped* 1,000 samples, trim portion set to 0.2). Participants who labeled the scowling faces as angry perceived the faces as less *Honest* [*Yuen’s bootstrapped t*(128) = −2.87, *p* = 0.005, *MD* = −0.301] and more *Threatening* [*Yuen’s bootstrapped t*(111) = 7.65, *p* < 0.001, *MD* = 0.89] than those who labeled the faces as disgusted.

## General Discussion

The data support the hypotheses that both the facial expression itself and the emotional interpretation of a facial expression guide the direction of the personality, temperament, and some social perception trait inferences. In our study, negative valence was demonstrated by negative inferences made to scowling faces, contrasting to the positive valence, and positive inferences made to smiling faces. Furthermore, participants who perceived the scowling faces as either angry or disgusted all made negative inferences, indicating that the expression itself creates negative valence in the perceiver, supporting Hypothesis 1 (scowling faces will initiate negative macro-valence and overall negative inferences compared to smiling faces, regardless of the emotion label assigned to the faces) and indicating support for the macro-valence concept. Participants labeling the scowling faces as angry inferred greater negative inferences while participants who perceived the same scowling faces as disgusted made less negative inferences, supporting Hypothesis 2 [that the different emotion labels (micro-valences) made to the scowling faces will result in differences in the negativity of inferences] and lending support for the micro valence concept.

Hypothesis 3 (that self-reported arousal ratings would be high for smiling faces labeled as happy and higher for scowling faces labeled as angry, rather than disgusted) was also supported. These results support the V-shaped model of the relation of arousal to valence proposed by Kuppens et al. (2013). The results also suggest that arousal is a component in both macro-valence assessment of a facial expression, and in the micro valence assessments leading to an emotion label of the facial expression. This is not surprising given that arousal is one of core components of both emotional expression and perception.

Of the four social perceptions, the two variables that failed to yield significant emotion label differences, *Attractiveness* and *Facial Maturity*, deserve explanation. Previous research exploring the effects of gender and age on the inference process showed that male faces were perceived as less *Facially*. *Mature* and less *Attractive* than female faces (Radeke and Stahelski, 2020). Additionally, and as expected, older models were perceived as more *Facially Mature* and less *Attractive* than younger faces (Radeke and Stahelski, 2020). We speculate that the results of this study, which combined age and gender, reflect a combination of the age and gender effects, resulting in the two non-significant results.

Additional support for Hypotheses 1 and 2 comes from research that examines behavioral reactions to perceived facial expressions. Mirabella (2018) examined participant reactions to happy faces versus fearful faces. Reactions are positive to happy faces and negative to fearful faces only if participants are focused on the emotions shown in the faces, as opposed to focusing on the gender of the individual in the pictured face. This finding demonstrates one, the positive/negative macro-valence of happy versus fearful faces, and two, the influence of the micro-valent appraisal of situational goal relevance.

Mancini et al. (2020), using the same research paradigm, compared reactions to angry faces, in addition to happy and fearful faces. They found that threatening faces (angry or fearful) increased reaction times and errors more than non-threatening faces when the facial expressions were relevant to the behavioral choice, and that angry faces increased reaction times and errors much more than fearful faces. Participants presented with angry faces presumably viewed them as direct threats, a micro-valent goal/need relevance appraisal correlated with increased arousal. Fearful faces, like faces labeled as disgust, may indicate a different micro-valent goal/need appraisal – that there is a possible indirect threat somewhere in the surrounding environment, an appraisal associated with reduced arousal. These results are similar to our results comparing anger and disgust labeling. Their measure of valence indicated that happy faces were rated positively and both threatening faces were rated negatively, supporting the macro-valence concept, and the different evaluations of the angry and fearful faces support the micro valence concept.

### Limitations and Further Directions

One obvious limitation of this study is the absence of a comparison of the other two negative facial expressions identified by Ekman and Friesen (1971): fearful, and sad. While the effect of emotion labeling on the social perceptions, temperament, and personality traits clearly demonstrates the importance of emotion labels in the facial inference process, an additional exploration of the comparison of incorrectly labeled scowling faces as either “fearful,” “sad,” or even “surprise” may serve to shed further light on the extent to which different negative labels to the same faces influence these inferences. Presumably, the same range of negative labels will occur with the other negative faces. Will the differing emotion labels to these other negative facial expressions lead to varying negative patterns of social, personality, and temperament inferences?

An additional limitation of this study is the absence of the neutral face as a comparison. Analyses of neutral faces labeled as anger and disgust (both experimenter labeled and participant labeled) might or might not support the findings of this study; consequently, the use of neutral faces as comparisons would provide further explanation of the inference process. Finally, smiling faces could be presented with variations in the obviousness of the smile. It is possible that variations in smiling would lead to variations in positive emotion labeling, which would lead to variations in the positivity of trait inferences.

### Theoretical Conclusion

Research reviewed in the Introduction section of this article indicate that emotion words used as labels influence the emotional inferences made to various facial expressions (Gendron et al., 2012; Fugate et al., 2018). The results from this study demonstrate that the influence of emotion labels goes beyond emotional inferences. Personality, temperament, and social trait inferences are influenced by different emotion labels made to the same facial expressions. From the perceiver’s viewpoint, facially expressed emotions can signal the perceived person’s intentions (Keltner and Cordaro, 2017). Facially expressed emotions can also signal the possible presence of various internal characteristics and traits within the perceived person that may relate to that person’s emotional and behavioral intentions. Perhaps, the emotional label becomes a heuristic signal that leads to a suite of inferences that the perceiver has learned to associate with specific emotions.

Possibly, the emotion label is a stereotype indicator, serving as a “trigger” in the minds of perceivers, bringing up a preconceived list of attributes and traits long associated with the specific emotive labeling of facial expressions (Knutson, 1996). Humans begin reading emotions from the faces of others in infancy (Frank et al., 2014) and they begin stereotyping in infancy (Serbin et al., 2001). By adulthood, we all have a great deal of experience attaching emotion labels to facial expressions. We also have experienced how those whose facial expressions we have labeled behave in various contexts. Humans develop a schema of characteristics and traits that presumably account for the behavior we observe that we associate with a specific emotion label.

Our results appear to at least partially support Scherer’s levels of valence theory, if variations in arousal are added to the overall theory (Scherer, 1984; Shuman et al., 2013). Participants assessed smiling faces as positive and evaluated scowling faces as generally negative, as shown by their negative-only emotional, social, temperament, and personality inferences. This is a demonstration of what Scherer called macro-valence, a one-dimensional affective overview. Participants subdivided their overall reactions into different negative emotion labels, reflecting what Scherer referred to as micro-valences.

In the 2013 article, Shuman et al. state that macro- and micro-valences and emotions occur simultaneously in an individual. However, Panksepp and Watt’s (2011) multi-level neurological conceptualization of emotional experience postulates a sequence. The first level of the sequence is primitive, in which basic affective feelings are generated in the sub-cortical regions of the brain. The authors state that the primitively generated feelings are either positive or negative, echoing Scherer’s macro-valence concept. At the second level (cortical level), the primitive feelings are shaped and conditioned by individual, environmental and cultural factors encapsulated in different micro-valences that lead to different emotion labels varying in arousal.

## Summary

The emotion labeling of facial expressions is an important part of the facial inferencing process. We believe that the results presented here increase the overall understanding of when and how emotion labeling influences facial inferencing, and our understanding of the limitations of emotion labeling. Unlabeled facial expressions act as stimuli influencing the perception of emotional valence. The emotion labels of expressions varying in arousal cause shifts within overall negative valence.

## Data Availability Statement

The raw data supporting the conclusions of this article will be made available by the authors, without undue reservation.

## Ethics Statement

The studies involving human participants were reviewed and approved by the Central Washington University, Human Subjects Review Council, https://www.cwu.edu/hsrc/. Authors are students or faculty at Central Washington University. Written informed consent for participation was not required for this study in accordance with the national legislation and the institutional requirements.

## Author Contributions

AS and MR contributed to the conception and design of the study and collected the data. AA, MR, and NB contributed to the data analysis. AS, MR, and AA contributed to the manuscript construction and editing. All authors contributed to the article and approved the submitted version.

## Funding

Funding for this research was provided by the Central Washington University Provost Faculty/Student Research Grant 2020-2021.

## Conflict of Interest

The authors declare that the research was conducted in the absence of any commercial or financial relationships that could be construed as a potential conflict of interest.

## Publisher’s Note

All claims expressed in this article are solely those of the authors and do not necessarily represent those of their affiliated organizations, or those of the publisher, the editors and the reviewers. Any product that may be evaluated in this article, or claim that may be made by its manufacturer, is not guaranteed or endorsed by the publisher.
