# Corrigendum: Facial expressions and emotion labels are separate initiators of trait inferences from the face

**DOI:** 10.3389/fpsyg.2022.1095086

**Published:** 2022-12-07

**Authors:** Anthony Stahelski, Amber Anderson, Nicholas Browitt, Mary Radeke

**Affiliations:** ^1^Department of Psychology, Central Washington University, Ellensburg, WA, United States; ^2^Department of Psychology, Universitetet i Oslo, Oslo, Norway

**Keywords:** facial expressions, emotion labels, facial inferencing, valence, personality

In the published article, there was an error in Figure 1. The wrong images were used in the original figure. The corrected Figure 1 and its updated caption are shown below.

**Figure 1:**
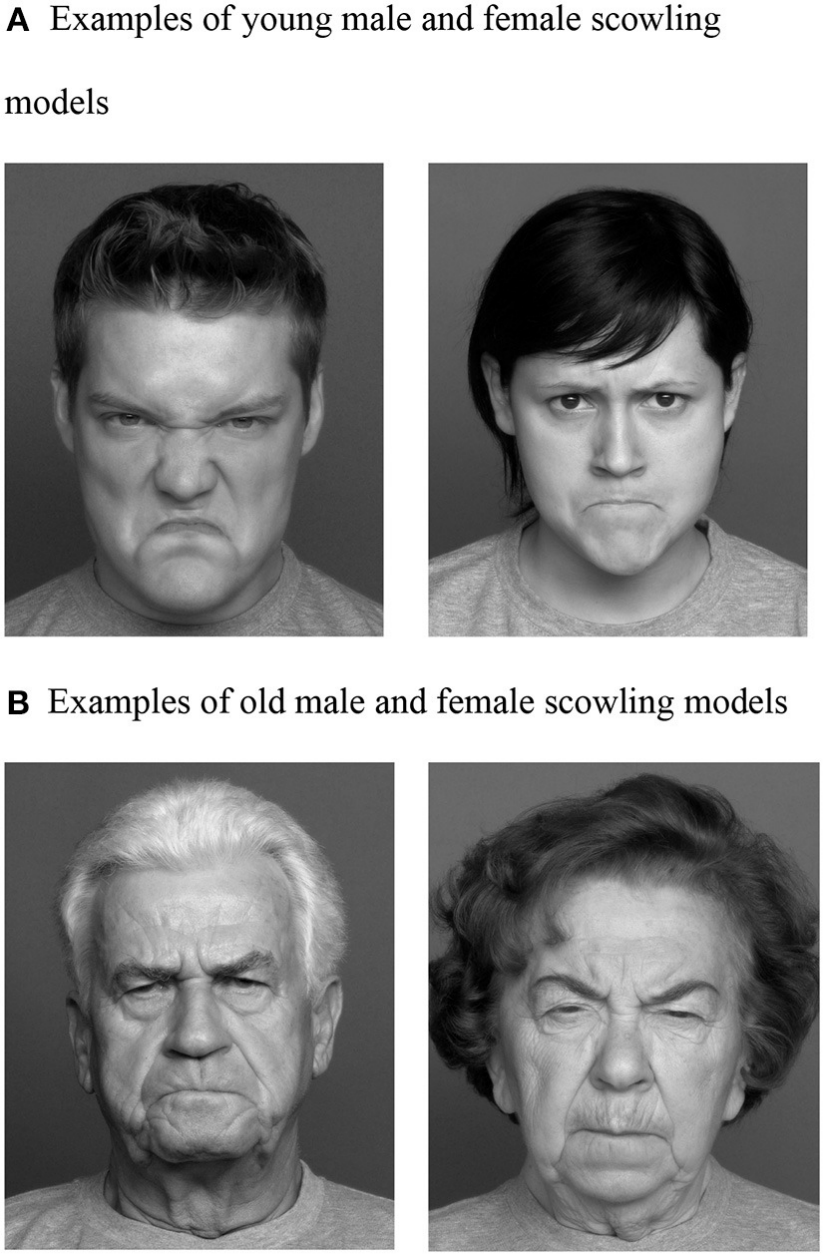
Facial photographs (Ebner et al., 2010) the Center for Lifespan Psychology, Max Planck Institute for Human Development, Berlin, Germany. Used with permission from the Max Planck Institute. These models are examples of images used in this study and not the actual images used. **(A)** Examples of young male and female scowling models. **(B)** Examples of old male and female scowling models.

The authors apologize for this error and state that this does not change the scientific conclusions of the article in any way. The original article has been updated.

## Publisher's note

All claims expressed in this article are solely those of the authors and do not necessarily represent those of their affiliated organizations, or those of the publisher, the editors and the reviewers. Any product that may be evaluated in this article, or claim that may be made by its manufacturer, is not guaranteed or endorsed by the publisher.
